# Impact of Genital Infections and Antibiotic Use on Incidence of Preterm Birth: A Retrospective Observational Study

**DOI:** 10.3390/antibiotics13030240

**Published:** 2024-03-05

**Authors:** Daniela Teodora Marti, Felix Bratosin, Ovidiu Rosca, Roxana Folescu, Cosmin Citu, Adrian Ratiu, Zoran Laurentiu Popa

**Affiliations:** 1 Clinical Analysis Laboratory, Emergency Clinical Hospital of Arad County, 310037 Arad, Romania; marti.teodora@uvvg.ro; 2 Department of Biology and Life Sciences, Vasile Goldis University of Medicine, 310048 Arad, Romania; 3 Department of Infectious Diseases, “Victor Babes” University of Medicine and Pharmacy Timisoara, 300041 Timisoara, Romania; ovidiu.rosca@umft.ro; 4 Doctoral School, “Victor Babes” University of Medicine and Pharmacy Timisoara, 300041 Timisoara, Romania; 5 Methodological and Infectious Diseases Research Center, Department of Infectious Diseases, “Victor Babes” University of Medicine and Pharmacy Timisoara, 300041 Timisoara, Romania; 6 Department of Family Medicine, “Victor Babes” University of Medicine and Pharmacy Timisoara, 300041 Timisoara, Romania; folescu.roxana@umft.ro; 7 Department of Obstetrics and Gynecology, “Victor Babes” University of Medicine and Pharmacy Timisoara, 300041 Timisoara, Romania; citu.ioan@umft.ro (C.C.); ratiu.adrian@umft.ro (A.R.); popa.zoran@umft.ro (Z.L.P.)

**Keywords:** genital infections, preterm birth, obstetric outcomes

## Abstract

This study investigates the complex interplay among genital infections, antibiotic usage, and preterm birth. This study aims to identify common genital pathogens associated with preterm births, assess the impact of various antibiotic treatments on pregnancy outcomes, and understand antibiotic resistance patterns among these pathogens. This study included 71 pregnant women who experienced preterm birth and 94 women with genital infections who delivered at term. Various maternal characteristics, medical history, signs and symptoms, gestational weight, gestational age, type of birth, vaginal pH, Nugent scores, and vaginal flora were analyzed. Antibiotic resistance patterns of isolated microorganisms were also examined. The prevalence of sexually transmitted diseases (STDs) and genital herpes was significantly higher in the preterm group. Preterm births were associated with fever, pelvic pain, vaginal spotting, and fatigue. Vaginal pH levels and Nugent scores were significantly higher in the preterm group, indicating disturbed vaginal flora. The presence of Extended-Spectrum Beta-Lactamases (ESBLs) was a particularly strong risk factor, increasing by more than four times the odds of preterm birth (OR = 4.45, *p* = 0.001). Vancomycin-Resistant Enterococci (VRE) presence was another critical factor, with a four-fold increase in the odds of preterm birth (OR = 4.01, *p* = 0.034). The overall presence of Multidrug-Resistant (MDR) organisms significantly increased the odds of preterm birth (OR = 3.73, *p* = 0.001). Specific pathogens like *Chlamydia trachomatis* (OR = 3.12, *p* = 0.020) and *Mycoplasma hominis* (OR = 3.64, *p* = 0.006) were also identified as significant risk factors. *Ureaplasma urealyticum* also showed a significantly higher risk of preterm birth (OR = 2.76, *p* = 0.009). This study highlights the importance of screening for and treating genital infections during pregnancy, especially STDs and genital herpes, as they can significantly increase the risk of preterm birth. Additionally, the presence of specific microorganisms and antibiotic resistance patterns plays an essential role in preterm birth risk. Early detection and targeted antibiotic treatment may help mitigate this risk and improve pregnancy outcomes.

## 1. Introduction

Preterm birth, defined as delivery before 37 weeks of gestation, remains a significant public health challenge globally, as approximately 15 million babies are born preterm every year, with an average of 10% of all live births, depending on regional development [1,2]. It is a leading cause of neonatal mortality and long-term morbidity, with profound implications for families and healthcare systems [3,4]. Various risk factors have been identified in the etiology of preterm birth, including demographic, genetic, environmental, and, particularly, infectious contributors [5,6,7,8,9,10,11]. Among these, genital infections in pregnant women are recognized as critical factors that can disrupt the normal course of gestation and precipitate early labor [12,13].

Genital infections, encompassing a range of bacterial, viral, and fungal pathogens, can ascend from the lower genital tract to affect the uterus, amniotic fluid, and fetus [8,9]. The inflammatory response triggered by these infections, as well as systemic inflammation are well-documented causes of preterm labor [14,15]. Bacteria such as Group B *Streptococcus*, *Trichomonas vaginalis*, and bacterial vaginosis-associated organisms are frequently implicated by inducing the release of pro-inflammatory cytokines, prostaglandins, and other mediators that lead to cervical ripening, uterine contractions, and eventual premature rupture of membranes [16,17,18,19,20,21,22].

However, the management of these infections during pregnancy is complicated by the need to consider both maternal and fetal safety. Antibiotics are the primary line of defense against bacterial genital infections, but their use during pregnancy is a delicate balance between eradicating the infection and preserving the health of both mother and fetus [23,24]. The choice of antibiotic, timing, and duration of treatment are critical factors that need careful consideration. Moreover, the emergence of antibiotic-resistant pathogens adds another layer of complexity to the management of these infections [25,26,27,28].

Despite the known association between genital infections and preterm birth, there are gaps in our understanding of the mechanistic pathways and the effectiveness of different antibiotic treatments in preventing preterm birth. Additionally, there is a need to understand the role of antibiotic stewardship in this context to prevent the overuse of antibiotics and the subsequent rise in resistant pathogens [29,30,31,32]. These gaps underscore the importance of continued research in this area to develop targeted strategies for the prevention and management of genital infections in pregnant women.

Considering the above, this study aims to explore the multifaceted relationship among genital infections, antibiotic use, and preterm birth. We hypothesize that certain genital infections significantly increase the risk of preterm birth, and that appropriate and timely antibiotic treatment can mitigate this risk. The objectives include identifying the most common genital pathogens implicated in preterm births, assessing the impact of different antibiotic therapies on pregnancy outcomes, and understanding the patterns of antibiotic resistance among these pathogens.

## 2. Results

In the current study, a total of 71 pregnant women were selected based on their status of genital infections during pregnancy and the preterm birth outcome, and another group of 94 pregnant women with genital infections who gave birth at term were also included. The mean age of women who gave birth preterm was 27.3 years, ranging from 18 to 40 years, while the mean age for the group of full-term births was slightly higher (28.1 years). However, this difference was not statistically significant (*p* = 0.341). Similarly, the distribution of age categories, with 62.0% of births occurring in women under 35 years in the preterm group and 54.3% in the full-term group, did not show a significant difference (*p* = 0.320).

In the preterm group, obesity was found in 16.9% of individuals, and this was slightly higher in the full-term group (19.1%), although this difference was not statistically meaningful (*p* = 0.710). Smoking during pregnancy occurred more frequently in the preterm group, 18.3%, compared with the full-term group, 11.7% (*p* = 0.233). Additionally, alcohol consumption during pregnancy was nearly the same between the preterm (11.3%) and full-term (10.6%) groups, with no statistically significant difference noted (*p* = 0.897). Regarding parity, the proportion of primigravida was slightly lower in the preterm group (53.5%) compared with the full-term group (57.4%). The proportion of multigravida women was correspondingly higher in the preterm group (46.5%) compared with the full-term group (42.6%), but these differences were not statistically significant (*p* = 0.615).

In the medical history of the participants, this study found a significantly higher prevalence of sexually transmitted diseases (STDs) in the preterm group (25.4%) compared with the full-term group (3.2%), which was statistically significant (*p* < 0.001). Genital herpes also showed a higher prevalence in the preterm group (8.5%) compared with the full-term group (1.1%), a difference that was statistically significant (*p* = 0.019), as presented in Table 1. However, no significant differences were observed in the prevalence of other conditions, such as urinary tract infections (UTIs), hypertension, diabetes, anemia, respiratory infections during pregnancy, and diarrheal illnesses.

The prevalence of fever was notably higher in the preterm group, with 81.7% of women experiencing fever compared with only 25.5% in the full-term group, a difference that was statistically significant (*p* < 0.001). Pelvic pain was also more common in the preterm group, affecting 50.7% of the women, as opposed to 21.3% in the full-term group, another statistically significant finding (*p* < 0.001). Other symptoms, such as foul-smelling vaginal discharge, genital itching, and urinary frequency, though more prevalent in the preterm group, did not show statistically significant differences. The prevalence rates of foul-smelling vaginal discharge were 45.1% in the preterm group and 37.2% in the full-term group (*p* = 0.310), genital itching was reported by 66.2% of the preterm group compared with 55.3% of the full-term group (*p* = 0.158), and urinary frequency was observed in 26.8% of the preterm group against 36.2% in the full-term group (*p* = 0.199).

Vaginal spotting was another symptom that showed a statistically significant difference, with 40.8% of the preterm group experiencing it against 24.5% of the full-term group (*p* = 0.024). However, symptoms like dyspareunia, nausea, headache, dizziness, and muscle cramps, though varied in prevalence between the two groups, did not reach statistical significance. Fatigue was significantly more prevalent in the preterm group, affecting 59.2% of the women, compared with 36.2% in the full-term group, a difference that was statistically significant (*p* = 0.003), as described in Table 2.

A key finding was the statistically significant difference in gestational weight between the two groups (*p* = 0.010). In the preterm group, infants with a gestational weight of 500–999 g and 1000–1499 g were represented by 2.8% and 8.5% respectively, whereas there were no infants in these weight categories in the full-term group. For infants weighing between 1500 and 2499 g, the prevalence was relatively similar in both groups (21.1% in the preterm group and 22.3% in the full-term group). Notably, a higher proportion of infants in the full-term group (77.7%) had a gestational weight of over 2500 g compared with the preterm group (67.6%).

This study also delved into gestational age categories, highlighting a stark contrast between preterm and full-term deliveries. Early preterm births (<28 weeks) constituted 11.3% of the preterm group, with no occurrences in the full-term group. Similarly, moderate preterm (28–32 weeks) and later preterm (32–36 weeks) births were exclusively observed in the preterm group, accounting for 46.5% and 42.3%, respectively. In contrast, early term (37–38 weeks) births occurred only in the full-term group (21.3%), as did full-term (38–42 weeks) and post-term (>42 weeks) births, constituting 70.2% and 8.5% of the full-term group, respectively.

Regarding the type of birth, a significant difference was observed (*p* < 0.001). Vaginal births were more common in the full-term group (73.4%) compared with the preterm group (39.4%). Cesarean births were more prevalent in the preterm group (56.3%) than in the full-term group (20.2%). The rate of assisted births was similar in both groups, with 4.2% in the preterm group and 6.4% in the full-term group (Table 3).

A key observation was the significant difference in vaginal pH levels between the two groups. The average pH level in the preterm group was 5.5, notably higher than the 4.5 observed in the full-term group, with this difference reaching statistical significance (*p* = 0.001). This study also evaluated the vaginal flora based on Nugent scores, finding significant differences in the prevalence of various flora types. The proportion of women with normal flora (Nugent scores of 0–3) was significantly lower in the preterm group (16.9%) compared with the full-term group (41.5%) (*p* = 0.003). Conversely, intermediate flora (Nugent scores of 4–6) was more common in the preterm group (49.3%) than in the full-term group (38.3%). Notably, the prevalence of bacterial vaginosis (Nugent scores of 7–10) was significantly higher in the preterm group (33.8%) compared with the full-term group (20.2%).

Regarding the findings from Gram staining, there was a significant decrease in the presence of *Lactobacillus* in the preterm group (50.7%) compared with the full-term group (72.3%), which was statistically significant (*p* = 0.004). The prevalence of *Gardnerella vaginalis* was also significantly higher in the preterm group (40.8%) than in the full-term group (22.3%) (*p* = 0.010), as described in Table 4. However, the differences in the prevalence of *Candida* spp., bacterial vaginosis combined with fungi, Gram-positive cocci, and Gram-negative bacilli between the two groups were not statistically significant.

The prevalence of *Bacillus* spp. and *Corynebacterium* spp. was slightly higher in the full-term group compared with the preterm group, but these differences were not statistically significant (*Bacillus* spp.: 7.0% in preterm vs. 8.5% in full-term groups, *p* = 0.727; *Corynebacterium* spp.: 8.5% in preterm vs. 10.6% in full-term groups, *p* = 0.638). Similarly, the presence of *Enterococcus* spp., *Escherichia coli*, *Haemophilus influenzae*, *Klebsiella* spp., *Proteus mirabilis*, *Staphylococcus aureus*, *Streptococcus* spp., and *Trichomonas vaginalis* did not show statistically significant differences between the two groups.

However, there were statistically significant differences noted in the frequency of specific pathogens. The incidence of *Chlamydia trachomatis* was notably higher in the preterm group, 12.7%, versus the full-term group, merely 3.2% (*p* = 0.020). In addition, the preterm group exhibited a substantially higher rate of infections by *Mycoplasma hominis* (16.9%) compared with only 4.3% in the other group (*p* = 0.006). The presence of *Neisseria gonorrhoeae* was also significantly elevated in the preterm group, 7.0%, in contrast to the full-term group, with a significant difference (*p* = 0.042). Furthermore, *Ureaplasma urealyticum* was found to be much more common in the preterm group, with a rate of 19.7%, compared with 6.4% in the full-term group (*p* = 0.009), as seen in Table 5.

There was a much higher rate of ESBL presence in the preterm group (21.1%) compared with the full-term group (5.3%) (*p* < 0.001). In addition, VRE infections were found to be significantly more common in the preterm group (8.5%) than in the full-term group (1.1%, *p* = 0.034). Likewise, CRE infections were detected particularly in the preterm group (5.6%), with no presence in the full-term group (*p* = 0.022). Furthermore, the overall prevalence of MDR pathogens was considerably higher in the preterm group (28.2%) versus the full-term group (7.4%) (*p* < 0.001).

However, the prevalence of Methicillin-Resistant *Staphylococcus aureus* (MRSA) did not differ significantly between the preterm and full-term groups (2.8% vs. 3.2%, *p* = 0.891). Additionally, the resistance patterns for antibiotics such as Nitrofurantoin, Ampicillin/Sulbactam, Macrolides, Piperacillin/Tazobactam, Glycopeptides, 4th-Generation Cephalosporin, Ticarcillin/Clavulanic, and Quinolones were not significantly different between the two groups. Of 27 samples resistant to macrolides, 12 (40.7%) were Chlamydia and Mycoplasma.

On the other hand, a significant difference was observed in the resistance to Penicillin (42.3% in preterm group vs. 19.1% in full-term group, *p* = 0.001) and 2nd- and 3rd-Generation Cephalosporins. The rates of resistance to 2nd-Generation Cephalosporin were 29.6% in the preterm group and 12.8% in the full-term group (*p* = 0.011), and for 3rd-Generation Cephalosporin, they were 31.0% in the preterm group and 11.7% in the full-term group (*p* < 0.001), as described in Table 6.

The history of sexually transmitted diseases (STDs) was a notable risk factor, with an odds ratio of 2.28, indicating more than double the odds of preterm birth, which was statistically significant (*p* = 0.001). Genital herpes did not emerge as a significant risk factor, nor did fever during pregnancy. Vaginal spotting was similarly significant, increasing the odds by over two times (OR = 2.23, *p* = 0.024). Fatigue was also associated with an increased risk of preterm birth (OR = 2.09, *p* = 0.003).

**Figure 1:**
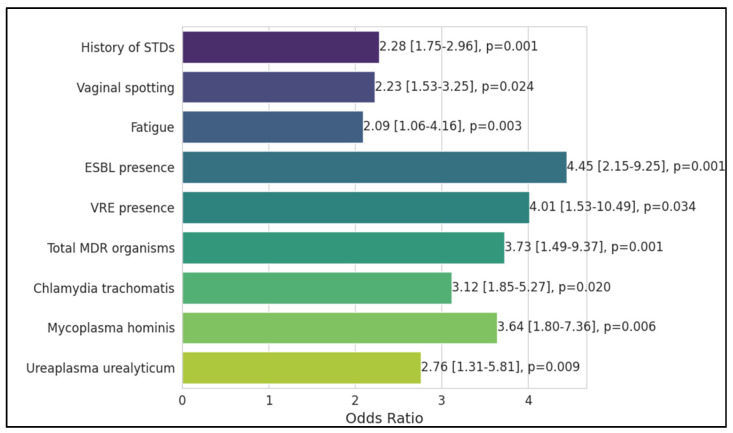
Significant risk factors associated with preterm birth.

The presence of Extended-Spectrum Beta-Lactamases (ESBLs) was a particularly strong risk factor, more than quadrupling the odds of preterm birth (OR = 4.45, *p* = 0.001). Vancomycin-Resistant Enterococci (VRE) presence was another critical factor, with a four-fold increase in the odds (OR = 4.01, *p* = 0.034). The overall presence of Multidrug-Resistant (MDR) organisms significantly increased the odds of preterm birth (OR = 3.73, *p* = 0.001). Specific pathogens like *Chlamydia trachomatis* (OR = 3.12, *p* = 0.020) and *Mycoplasma hominis* (OR = 3.64, *p* = 0.006) were identified as significant risk factors. *Ureaplasma urealyticum* also showed a significant association with increased odds of preterm birth (OR = 2.76, *p* = 0.009), as presented in Table 7 and Figure 1.

## 3. Discussion

### 3.1. Literature Findings

Our study findings, while highlighting the role of genital infections in preterm births, also suggest that the relationship between these infections and pregnancy outcomes is more complex than previously understood. In the current study, certain infections, such as *Chlamydia trachomatis*, *Mycoplasma hominis*, and *Ureaplasma urealyticum*, were more prevalent in the preterm group, as previously described in a meta-analysis [33]. However, another study’s findings [34] suggest that while these infections are associated with preterm births, they might not be the only determinants of adverse neonatal outcomes.

Moreover, this study’s findings regarding the prevalence of MDR organisms such as ESBLs and VRE in the preterm group underscore the complexities of treating infections during pregnancy. However, the specific impact of MDR bacteria on neonatal outcomes was not distinctly established, indicating the need for further research to understand the nuances of antibiotic resistance in the context of preterm births.

It is also important to consider other factors contributing to preterm births, such as maternal health conditions, lifestyle habits, and socioeconomic status. While this study observed a higher prevalence of fever and vaginal spotting in the preterm group, these symptoms alone are not enough to explain the complexities of preterm labor. It is plausible that a combination of these symptoms, along with underlying maternal health conditions and environmental factors, contribute to the incidence of preterm births.

The findings also reveal the complexity of diagnosing and managing maternal infections during pregnancy. The role of asymptomatic or subclinical infections and even maternal colonization by certain pathogens without evident infection cannot be overlooked. This complexity underscores the importance of comprehensive prenatal care, encompassing not just the treatment of identified infections but also a broader focus on maternal health and preventive strategies to mitigate the risk of preterm labor.

When comparing the findings of our study with those from a Brazilian multicentric study on preterm births (PTBs), several contrasts and parallels become evident, particularly in the prevalence and impact of maternal infections on PTBs [35]. In our study, specific genital infections and their antibiotic resistance patterns were highlighted as significant factors contributing to preterm births. On the other hand, the Brazilian study, encompassing 2682 women, found that a substantial majority (65.9%) had at least one maternal infection, with the majority being urinary tract infections and genital infections, which were reported by over half of the participants. Additionally, the Brazilian study highlighted the role of sociodemographic factors, noting that the presence of a partner was more common among women with infectious diseases (OR 1.28, *p* = 0.026). This aspect was not explored in our study, suggesting that the interplay between medical and social factors in the context of PTB is complex and multifaceted. These differences highlight the importance of a comprehensive approach to PTB management, considering both the clinical treatment of infections and broader sociodemographic influences [36].

In the review by Daskalakis et al. [36], several key similarities and differences in the understanding of the role of infections in preterm births emerged. Both their study and ours recognized the significant impact of infections on preterm birth, but the subject was approached from different perspectives. Our study specifically identified and focused on the prevalence of certain genital pathogens, such as *Chlamydia trachomatis*, *Mycoplasma hominis*, *and Ureaplasma urealyticum*, and their link to preterm births [33]. This is in line with Daskalakis et al.’s review, which also acknowledged these pathogens as contributors to premature delivery and related complications, such as chorioamnionitis and neonatal sepsis.

While our study delved into the nuances of antibiotic resistance patterns, it was found that of 27 samples resistant to macrolides, 12 (40.7%) were *Chlamydia* and *Mycoplasma*. Daskalakis et al. emphasized the broader mechanism of infection-induced inflammation leading to preterm birth. They highlighted the overproduction of prostaglandins due to infection-related inflammation as a key factor in triggering uterine contractions and subsequent preterm delivery. This difference in focus brings to light the multifaceted nature of infection-related preterm births, combining specific microbial involvement with the broader physiological responses to infection.

Another recent study highlighted a significant prevalence of *Chlamydia trachomatis* infections among young pregnant women below 25 years old, with an 18.4% infection rate and a 35% perinatal transmission rate. Therefore, the debate over the necessity of antenatal *Chlamydia* screening programs still remains [37]. Despite the demonstrated association between *Chlamydia* (and potentially *Mycoplasma*) infection and preterm birth, the effectiveness and requirement of such screening programs in preventing adverse birth outcomes are subjects of ongoing discussion. This controversy, as noted by Dorado CM et al. [37], questions the universal application of these programs across different healthcare settings, especially when considering the varying success rates in several developed countries.

Chelkeba et al.’s study [38] revealed a substantial prevalence of bacteriuria among pregnant women, with an overall pooled estimate of 15% and with *Escherichia coli* being the most common pathogen (41%). This high incidence parallels our study’s findings on the prevalence of specific bacterial infections. Importantly, Chelkeba et al. reported an alarming rate of multidrug resistance, with rates as high as 83% for *Escherichia coli* and 89% for *Staphylococcus aureus*. This aligns with our study’s emphasis on the challenge posed by antibiotic resistance in effectively managing infections during pregnancy.

Nguyen et al.’s study [39] provided a different perspective, assessing the impact of gestational antibiotic use on preterm birth risks. Their findings show an increased risk of preterm birth associated with antibiotic use during pregnancy, particularly in the first and second trimesters. The increased risks were notable for several antibiotic groups, such as macrolides, lincosamides, and streptogramins (OR = 1.63), and quinolones (OR = 1.60). In contrast, pre-conception antibiotic use showed no association with preterm births. These findings highlight the potential risk factors associated with antibiotic use during critical periods of pregnancy, underscoring a complex interplay between infection treatment and pregnancy outcomes. Both studies, together with our research, stress the importance of cautious antibiotic use and targeted infection management in pregnant women to mitigate the risks of preterm births.

Similarly, Samarra et al.’s study [40] brings to light the critical issue of antibiotic resistance in the context of maternal and newborn health. The authors highlighted the risk of vertical transmission of antibiotic resistance from mothers to infants, particularly at birth, a pivotal moment for the infant’s exposure to potentially resistant microorganisms. This aspect connects with our study’s focus on the prevalence and resistance patterns of specific pathogens in preterm births, and Chelkeba et al.’s [38] and Nguyen et al.’s [39] findings on the widespread antimicrobial resistance and its implications during pregnancy. Together, these studies underscore the far-reaching impact of antibiotic resistance, from influencing preterm birth outcomes to shaping the neonatal microbiome, underlining the need for a comprehensive approach to managing infections during pregnancy and early life.

The systematic review by Tong et al. [41] offers substantial empirical evidence on how treating syphilis, chlamydia, and gonorrhea during pregnancy affects birth outcomes. Remarkably, administering treatment to pregnant women diagnosed with syphilis decreased the likelihood of preterm birth by 52%, stillbirth by 79%, and low birth weight by 50%. Additionally, treating chlamydia in pregnant women was associated with a 42% reduction in the risk of preterm birth and a potential 40% decrease in the risk of low birth weight. While these findings are valuable in the context of our study, it is important to acknowledge that the overall quality of evidence was considered low due to limited adjustment for potential confounding factors. Despite this, the consistent and substantial effects observed in Tong et al.’s study emphasize the potential benefits of timely detection and treatment of maternal infections in improving birth outcomes, aligning with the themes explored in our research. However, further research is needed to better understand the effects of antibiotic treatment for chlamydia and gonorrhea infections during pregnancy.

### 3.2. Study Limitations

Several limitations are inherent to our study. Firstly, the retrospective nature of the research design may introduce recall bias, as the data were collected based on medical records and participant recollections. This reliance on historical information may result in underreporting or incomplete data, potentially affecting the accuracy of our findings. Secondly, this study’s exclusion criteria, which excluded individuals with various pregnancy complications and chronic conditions, may limit the generalizability of our results to a broader population. Additionally, the absence of certain clinical data, such as detailed information on antibiotic dosages and treatment durations, may hinder a comprehensive analysis of the antibiotic treatments’ effects on birth outcomes. Furthermore, this study’s focus on a specific geographical region and healthcare setting may restrict the applicability of our findings to different populations and healthcare contexts. While our sample size was adequate for overall analyses, we note limitations in detecting significant associations within certain subgroups. Therefore, the applicability of our findings may be specific only to the studied geographic and demographic context. Lastly, while we conducted a thorough assessment of microbial profiles and antibiotic resistance patterns, the absence of detailed information on the specific antibiotics used by participants during pregnancy limits our ability to draw precise conclusions about the antibiotic–resistance relationship. Despite these limitations, our study provides valuable insights into the complex interplay among maternal genital infections, antibiotic treatments, and birth outcomes.

## 4. Materials and Methods

### 4.1. Study Design

The present research utilized a case–control design, gathering information from expectant mothers from the 2019 and 2023 period who presented with genital infections at the Obstetrics and Gynecology department of Clinical County Hospital, Timisoara, and private obstetrics clinics. The region covered by this study comprised about 300 thousand inhabitants from the western region of Romania, of which approximately 70 thousand women are of reproductive age. The hospital’s large patient base and the variety of cases encountered in both public and private healthcare settings offer a comprehensive overview of the prevalence and impact of genital infections in pregnant women within the region.

Ethical validation for this study was obtained from the hospital’s Institutional Review Board, in compliance with the principles outlined in the Declaration of Helsinki, with the approval being dated 31 March 2023 (code E-1853). A retrospective examination of the data was conducted, and all participants had previously given their informed consent for their personal and medical details to be utilized in investigations during their initial assessment while preserving confidentiality and privacy of the patients’ personal information.

### 4.2. Selection Criteria

All patients in this study had a diagnosis of genital infection during their pregnancy. The “preterm” group consisted of women who gave birth prematurely before 37 weeks of pregnancy and had documented antibiotic use during pregnancy. The “full-term” group comprised pregnant women who gave birth at term and documented antibiotic use. The criteria for inclusion involved (1) pregnant women aged 18 and older, (2) availability of comprehensive medical histories for the duration of this study, (3) agreement to data collection, (4) positive bacterial cultures during pregnancy for genital infections, and (5) antibiotic consumption history.

Other pregnant women were not included if they had complications such as preterm premature rupture of membranes (PPROM), preeclampsia, cervical insufficiency, multiple gestations, restricted fetal growth, placenta previa, placental abruption, placenta accreta, hypertension during pregnancy, or diabetes during pregnancy. Those with incomplete or absent medical records were also not eligible for this study. Additionally, those with pre-existing chronic conditions that might independently impact infections or the outcome of the pregnancy, such as autoimmune disorders, were not considered. Similarly, those who had taken corticosteroids during pregnancy were excluded. Patients with HIV were not included due to the possible confounding effect of a lower immune status.

### 4.3. Study Variables and Microbial Assessment

In this study, a detailed analysis was conducted on an array of variables to discern their influences on preterm and full-term pregnancies. Background maternal characteristics, including age and Body Mass Index (BMI), were assessed alongside lifestyle factors like smoking and alcohol use during pregnancy. Medical history variables such as obesity, parity, urinary tract infections, sexually transmitted diseases, genital herpes, hypertension, diabetes, anemia, and respiratory and diarrheal illnesses during pregnancy were meticulously examined for their potential impact on birth outcomes.

This study also rigorously evaluated signs and symptoms prevalent in pregnancies, such as fever, pelvic pain, vaginal discharge, itching, spotting, dyspareunia, urinary frequency, nausea, headache, dizziness, fatigue, and muscle cramps. Neonatal outcomes like gestational weight, age, and type of birth were analyzed. Additionally, vaginal smear results, including pH levels, Nugent scores [42], and the presence of various bacteria and fungi, were scrutinized. This study extended its analysis to vaginal culture results, focusing on microorganisms, antibiotic sensitivity, and multidrug resistance patterns, crucial to understanding infection management in pregnancies. 

This research concentrated on identifying various types of bacteria, both Gram-negative and Gram-positive. It also examined the presence of Multidrug-Resistant organisms and patterns of antibiotic resistance, including Extended-Spectrum Beta-Lactamases (ESBLs), Methicillin-Resistant *Staphylococcus aureus* (MRSA), Vancomycin-Resistant Enterococci (VRE), and Carbapenem-Resistant Enterobacteriaceae (CRE). Antibiotic susceptibility tests were conducted using the VITEK^®^ 2 [43] system (bioMérieux, Inc., Hazelwood, MO, USA), and the findings were evaluated based on established guidelines. *Chlamydia* and *Mycoplasma* were identified through nucleic acid amplification tests. Upon collection, samples were immediately refrigerated at 4 °C to preserve the integrity of the nucleic acids and transported to the laboratory within 2 h of collection for processing.

Regarding quality control measures, our laboratory adhered to strict protocols to ensure the accuracy and dependability of our findings. The VITEK^®^ 2 system, utilized for antibiotic susceptibility testing, underwent daily calibration and performance verification against known control strains to ensure its precision in determining microbial susceptibility patterns. Laboratory personnel received regular training sessions on the latest advancements in microbiological techniques and handling of the VITEK^®^ 2 system, including the interpretation of results according to the Clinical and Laboratory Standards Institute (CLSI) [44], which provided the recommendations and criteria to determine antimicrobial susceptibility for all cultured bacteria.

### 4.4. Statistical Analysis

To address missing data, our methodology was designed to minimize bias and maintain the integrity of our analyses. Data analysis utilized SPSS version 27 (IBM Corp., Chicago, IL, USA). For English grammar and spelling errors, ChatGPT version 3.5 by OpenAI (San Francisco, CA, USA) was used. To determine the sample size, a convenience sampling method was applied, targeting a 95% confidence level with a 5% margin of error and an 11% worldwide prevalence rate of preterm births [45], requiring at least 139 cases. Descriptive statistics summarized demographic and clinical data. Differences in the proportions of microbial species and resistance patterns between the groups were analyzed using either the Chi-square test or Fisher’s exact test, depending on the frequency assumptions. The Kolmogorov–Smirnov test was employed to assess data normality. For continuous data, comparisons between two means were made using the independent samples *t*-test, while the Mann–Whitney U-test was utilized for comparing medians. Recognizing the inherent limitations of statistical models, our study carefully considered the assumptions of logistic regression and the potential impact of non-random missing data. Logistic regression models identified factors independently associated with the risk of preterm birth, considering a *p*-value of less than 0.05 to be statistically significant.

## 5. Conclusions

In conclusion, this study elucidates the intricate relationship among genital infections, antibiotic use, and the risk of preterm birth. Our findings underscore the significance of several key factors in influencing pregnancy outcomes. Notably, the presence of sexually transmitted diseases, particularly *Chlamydia trachomatis* and *Mycoplasma hominis*, emerged as substantial risk factors for preterm birth. Genital herpes, fever during pregnancy, vaginal spotting, and fatigue were also identified as significant contributors to the increased odds of preterm birth. Furthermore, this study revealed a striking association between the presence of MDR organisms/ESBLs/VRE and the risk of preterm birth. These findings emphasize the critical importance of early detection, appropriate antibiotic therapy, and vigilant management of genital infections during pregnancy to mitigate the risk of preterm birth and improve maternal and neonatal outcomes. Further research is warranted to explore the mechanisms underlying these associations and to develop targeted interventions for at-risk populations.

## Figures and Tables

**Table 1:** Maternal characteristics and medical history in preterm and full-term pregnancies.

Variables	Preterm (*n* = 71)	Full Term (*n* = 94)	*p*-Value
Age (mean ± SD)	27.3 ± 5.5	28.1 ± 5.2	0.341
Age range	18–40	21–39	-
Age category			0.320
<35 years	44 (62.0%)	51 (54.3%)	
≥35 years	27 (38.0%)	43 (45.7%)	
Obesity (BMI ≥ 30 kg/m^2^)	12 (16.9%)	18 (19.1%)	0.710
Smoking during pregnancy	13 (18.3%)	11 (11.7%)	0.233
Alcohol use during pregnancy	8 (11.3%)	10 (10.6%)	0.897
Parity			0.615
Primigravida	38 (53.5%)	54 (57.4%)	
Multigravida	33 (46.5%)	40 (42.6%)	
ART-achieved pregnancies	6 (8.5%)	7 (7.4%)	0.812
Medical history			
UTIs	9 (12.7%)	14 (14.9%)	0.683
STDs	18 (25.4%)	3 (3.2%)	**<0.001**
Genital herpes	6 (8.5%)	1 (1.1%)	**0.019**
Hypertension	7 (9.9%)	9 (9.6%)	0.951
Diabetes	5 (7.0%)	10 (10.6%)	0.426
Anemia	8 (11.3%)	6 (6.4%)	0.264
Respiratory infections during pregnancy	11 (15.5%)	13 (13.8%)	0.764
Diarrheal illness during pregnancy	9 (12.7%)	12 (12.8%)	0.986
Thyroid disease	3 (4.2%)	1 (1.1%)	0.191
Others	6 (8.5%)	10 (10.6%)	0.638

BMI—Body Mass Index; SD—Standard Deviation; UTI—urinary tract infection; ART—Assisted Reproductive Technique; STD—Sexually Transmitted Disease. Bold values are statistically significant.

**Table 2:** Prevalence of signs and symptoms in preterm versus full-term pregnancies.

Signs and Symptoms (*n*, %)	Preterm (*n* = 71)	Full Term (*n* = 94)	*p*-Value
Fever	58 (81.7%)	24 (25.5%)	**<0.001**
Pelvic pain	36 (50.7%)	20 (21.3%)	**<0.001**
Foul-smelling vaginal discharge	32 (45.1%)	35 (37.2%)	0.310
Genital itching	47 (66.2%)	52 (55.3%)	0.158
Vaginal spotting	29 (40.8%)	23 (24.5%)	**0.024**
Dyspareunia	22 (31.0%)	26 (27.7%)	0.641
Urinary frequency	19 (26.8%)	34 (36.2%)	0.199
Nausea	35 (49.3%)	37 (39.4%)	0.202
Headache	20 (28.2%)	22 (23.4%)	0.486
Dizziness	7 (9.9%)	11 (11.7%)	0.706
Fatigue	42 (59.2%)	34 (36.2%)	**0.003**
Muscle cramps	17 (23.9%)	14 (14.9%)	0.140

Bold values are statistically significant.

**Table 3:** Neonatal outcomes in preterm and full-term deliveries.

Variables	Preterm (*n* = 71)	Full Term (*n* = 94)	*p*-Value
Gestational Weight			0.010
500–999 g	2 (2.8%)	0 (0.0%)	
1000–1499 g	6 (8.5%)	0 (0.0%)	
1500–2499 g	15 (21.1%)	21 (22.3%)	
>2500 g	48 (67.6%)	73 (77.7%)	
Gestational Age			–
Early preterm (<28 weeks)	8 (11.3%)	0 (0.0%)	
Moderate preterm (28–32 weeks)	33 (46.5%)	0 (0.0%)	
Later preterm (32–36 weeks)	30 (42.3%)	0 (0.0%)	
Early term (37–38 weeks)	0 (0.0%)	20 (21.3%)	
Full term (38–42 weeks)	0 (0.0%)	66 (70.2%)	
Post term (>42 weeks)	0 (0.0%)	8 (8.5%)	
Type of Birth			**<0.001**
Vaginal	28 (39.4%)	69 (73.4%)	
Cesarean	40 (56.3%)	19 (20.2%)	
Assisted	3 (4.2%)	6 (6.4%)	

Bold values are statistically significant.

**Table 4:** Vaginal smear results comparison for patients who gave birth preterm and at full term.

Variables	Preterm (*n* = 71)	Full Term (*n* = 94)	*p*-Value
pH test	5.5 (5.0–6.1)	4.5 (4.2–4.9)	**0.001**
Overall assessment (Nugent score)			**0.003**
0–3 (normal flora)	12 (16.9%)	39 (41.5%)	
4–6 (intermediate flora)	35 (49.3%)	36 (38.3%)	
7–10 (bacterial vaginosis)	24 (33.8%)	19 (20.2%)	
Gram staining			
*Lactobacillus*	36 (50.7%)	68 (72.3%)	**0.004**
*Gardnerella vaginalis*	29 (40.8%)	21 (22.3%)	**0.010**
*Candida* spp.	22 (31.0%)	27 (28.7%)	0.752
Bacterial vaginosis and fungi	7 (9.9%)	5 (5.3%)	0.266
Gram-positive cocci	18 (25.4%)	9 (9.6%)	**0.007**
Gram-negative bacilli	6 (8.5%)	3 (3.2%)	0.140

*Gardnerella vaginalis* was identified as clue cells; *Candida* spp. were identified as budding yeast with pseudohyphae. Bold values are statistically significant.

**Table 5:** Vaginal culture results comparison for patients who gave birth preterm and at full term.

Variables	Preterm (*n* = 71)	Full Term (*n* = 94)	*p*-Value
*Bacillus* spp.	5 (7.0%)	8 (8.5%)	0.727
*Corynebacterium* spp.	6 (8.5%)	10 (10.6%)	0.638
*Chlamydia trachomatis*	9 (12.7%)	3 (3.2%)	**0.020**
*Enterococcus* spp.	10 (14.1%)	15 (16.0%)	0.739
*Escherichia coli*	9 (12.7%)	7 (7.4%)	0.281
*Haemophilus influenzae*	2 (2.8%)	5 (5.3%)	0.477
*Klebsiella* spp.	7 (9.9%)	6 (6.4%)	0.410
*Mycoplasma hominis*	12 (16.9%)	4 (4.3%)	**0.006**
*Neisseria gonorrhoeae*	5 (7.0%)	1 (1.1%)	**0.042**
*Proteus mirabilis*	4 (5.6%)	3 (3.2%)	0.496
*Staphylococcus aureus*	11 (15.5%)	9 (9.6%)	0.211
*Streptococcus* spp.	13 (18.3%)	18 (19.1%)	0.904
*Trichomonas vaginalis*	6 (8.5%)	5 (5.3%)	0.424
*Ureaplasma urealyticum*	14 (19.7%)	6 (6.4%)	**0.009**

Bold values are statistically significant.

**Table 6:** Multidrug resistance patterns and antibiotic sensitivity testing among pregnant women with genital infections, stratified by preterm and full-term births.

Variables	Preterm (*n* = 71)	Full Term (*n* = 94)	Total	*p*-Value
ESBLs	15 (21.1%)	5 (5.3%)	20/165 (12.1%)	**<0.001**
MRSA	2 (2.8%)	3 (3.2%)	5/165 (3.0%)	0.891
VRE	6 (8.5%)	1 (1.1%)	7/165 (4.2%)	**0.034**
CRE	4 (5.6%)	0 (0.0%)	4/165 (2.4%)	**0.022**
Total MDR	20 (28.2%)	7 (7.4%)	27/165 (16.4%)	**<0.001**
Penicillins	30 (42.3%)	18 (19.1%)	48/165 (29.1%)	**0.001**
Nitrofurantoin	14 (19.7%)	22 (23.4%)	36/165 (21.8%)	0.564
Ampicillin/Sulbactam	10 (14.1%)	13 (13.8%)	23/165 (13.9%)	0.956
Macrolides	12 (16.9%)	15 (16.0%)	27/165 (16.4%)	0.924
Piperacillin/Tazobactam	7 (9.9%)	9 (9.6%)	16/165 (9.7%)	0.973
Glycopeptides	11 (15.5%)	6 (6.4%)	17/165 (10.3%)	**0.043**
2nd-Gen. Cephalosporin	21 (29.6%)	12 (12.8%)	33/165 (20.0%)	**0.011**
3rd-Gen. Cephalosporin	22 (31.0%)	11 (11.7%)	33/165 (20.0%)	**<0.001**
4th-Gen. Cephalosporin	6 (8.5%)	10 (10.6%)	16/165 (9.7%)	0.660
Ticarcillin/Clavulanic	5 (7.0%)	8 (8.5%)	13/165 (7.9%)	0.760
Quinolones	3 (4.2%)	6 (6.4%)	9/165 (5.5%)	0.524

ESBLs—Extended-Spectrum Beta-Lactamases; MRSA—Methicillin-Resistant *Staphylococcus aureus*; VRE—Vancomycin-Resistant Enterococci; CRE—Carbapenem-Resistant Enterobacteriaceae; MDR—Multidrug Resistant. Bold values are statistically significant.

**Table 7:** Significant risk factors associated with preterm birth.

Significant Risk Factors	Coefficient (β)	SE	OR	95% CI	*p*-Value
History of STDs	0.82	0.13	2.28	1.75–2.96	**0.001**
Genital herpes	0.62	0.33	1.85	0.96–3.56	0.079
Fever	0.78	0.5	2.18	0.82–5.78	0.051
Vaginal spotting	0.8	0.19	2.23	1.53–3.25	**0.024**
Fatigue	0.74	0.35	2.09	1.06–4.16	**0.003**
ESBLs presence	1.49	0.37	4.45	2.15–9.25	**0.001**
VRE presence	1.39	0.49	4.01	1.53–10.49	**0.034**
Total MDR organisms	1.32	0.47	3.73	1.49–9.37	**0.001**
*Chlamydia trachomatis*	1.14	0.27	3.12	1.85–5.27	**0.020**
*Mycoplasma hominis*	1.29	0.36	3.64	1.80–7.36	**0.006**
*Ureaplasma urealyticum*	1.02	0.38	2.76	1.31–5.81	**0.009**

OR—odds ratio; CI—Confidence Interval; SE—Standard Error; STDs—sexually transmitted diseases; MDR—Multidrug-Resistant; ESBLs—Extended-Spectrum Beta-Lactamases; VRE—Vancomycin-Resistant Enterococci. Bold values are statistically significant.

## Data Availability

Data are available upon request.

## References

[1] Ayele T.B., Moyehodie Y.A. (2023). Prevalence of preterm birth and associated factors among mothers who gave birth in public hospitals of east Gojjam zone, Ethiopia. BMC Pregnancy Childbirth.

[2] Chawanpaiboon S., Vogel J.P., Moller A.B., Lumbiganon P., Petzold M., Hogan D., Landoulsi S., Jampathong N., Kongwattanakul K., Laopaiboon M. (2019). Global, regional, and national estimates of levels of preterm birth in 2014: A systematic review and modelling analysis. Lancet Glob. Health.

[3] Mocking M., Adu-Bonsaffoh K., Osman K.A., Tamma E., Ruiz A.M., van Asperen R., Oppong S.A., Kleinhout M.Y., Gyamfi-Bannerman C., Browne J.L. (2023). Causes, survival rates, and short-term outcomes of preterm births in a tertiary hospital in a low resource setting: An observational cohort study. Front. Glob. Womens Health.

[4] Simhan H.N. (2010). Preterm birth is the leading cause of neonatal mortality and is responsible for roughly one-half of long-term neurologic sequelae. Am. J. Obstet. Gynecol..

[5] Dahman H.A.B. (2020). Risk factors associated with preterm birth: A retrospective study in Mukalla Maternity and Childhood Hospital, Hadhramout Coast/Yemen. Sudan. J. Paediatr..

[6] Dohou A.M., Buda V.O., Anagonou S., Van Bambeke F., Van Hees T., Dossou F.M., Dalleur O. (2022). Healthcare Professionals’ Knowledge and Beliefs on Antibiotic Prophylaxis in Cesarean Section: A Mixed-Methods Study in Benin. Antibiotics.

[7] Turaiche M., Feciche B., Gluhovschi A., Bratosin F., Bogdan I., Bota A.V., Grigoras M.L., Gurban C.V., Cerbu B., Toma A.-O. (2022). Biological Profile and Clinical Features as Determinants for Prolonged Hospitalization in Adult Patients with Measles: A Monocentric Study in Western Romania. Pathogens.

[8] Lombrea A., Romanescu M., Jianu N., Andor M., Suciu M., Man D.E., Danciu C., Dehelean C.A., Buda V. (2023). Sex-Related Differences in the Pharmacological Response in SARS-CoV-2 Infection, Dyslipidemia, and Diabetes Mellitus: A Narrative Review. Pharmaceuticals.

[9] Bînă A.M., Aburel O.M., Avram V.F., Lelcu T., Lința A.V., Chiriac D.V., Mocanu A.G., Bernad E., Borza C., Craina M.L. (2022). Impairment of mitochondrial respiration in platelets and placentas: A pilot study in preeclamptic pregnancies. Mol. Cell Biochem..

[10] Oşvar F.N., Raţiu A.C., Voiţă-Mekereş F., Voiţă G.F., Bonţea M.G., Racoviţă M., Mekereş G.M., Bodog F.D. (2020). Cardiac axis evaluation as a screening method for detecting cardiac abnormalities in the first trimester of pregnancy. Rom. J. Morphol. Embryol..

[11] Hrubaru I., Motoc A., Bratosin F., Rosca O., Folescu R., Moise M.L., Neagoe O., Citu I.M., Feciche B., Gorun F. (2022). Exploring Clinical and Biological Features of Premature Births among Pregnant Women with SARS-CoV-2 Infection during the Pregnancy Period. J. Pers. Med..

[12] Kumar M., Saadaoui M., Al Khodor S. (2022). Infections and Pregnancy: Effects on Maternal and Child Health. Front. Cell Infect. Microbiol..

[13] Goldenberg R.L., Culhane J.F., Johnson D.C. (2005). Maternal infection and adverse fetal and neonatal outcomes. Clin. Perinatol..

[14] Stoicescu E.R., Ciuca I.M., Iacob R., Iacob E.R., Marc M.S., Birsasteanu F., Manolescu D.L., Iacob D. (2021). Is Lung Ultrasound Helpful in COVID-19 Neonates?-A Systematic Review. Diagnostics.

[15] Chan M.Y., Smith M.A. (2018). Infections in Pregnancy. Comprehensive Toxicology.

[16] Gobjila C., Craina M.L., Toader D.O., Petre I., Andor C.B., Tudor A., Onofrei R.R., Tamas L.A., Ilie A.C. (2019). Pro-inflammatory Cytokines (IL6, IL8 and TNF-a) in the Evaluation of Ovarian Endometriosis Cyst. Rev. Chim..

[17] Bagga R., Arora P. (2020). Genital Micro-Organisms in Pregnancy. Front. Public. Health.

[18] Romero R., Espinoza J., Gonçalves L.F., Kusanovic J.P., Friel L., Hassan S. (2007). The role of inflammation and infection in preterm birth. Semin. Reprod. Med..

[19] Prodan-Barbulescu C.F., Faur F.I., Stoica L., Isaic A., Clim A., Nati I., Dobrescu A. (2022). Differences among Obese versus Nonobese Patients undergoing total Laparoscopic Hysterectomy. A single Center Experience. J. Clin. Res. Rep..

[20] Mirmonsef P., Krass L., Landay A., Spear G.T. (2012). The role of bacterial vaginosis and trichomonas in HIV transmission across the female genital tract. Curr. HIV Res..

[21] Ionut Flaviu F., Clim A., Dobrescu A., Prodan C., Hajjar R., Pasca P., Capitanio M., Tarta C., Isaic A., Noditi G. (2023). VRAM Flap for Pelvic Floor Reconstruction after Pelvic Exenteration and Abdominoperineal Excision. J. Pers. Med..

[22] Redelinghuys M.J., Geldenhuys J., Jung H., Kock M.M. (2020). Bacterial Vaginosis: Current Diagnostic Avenues and Future Opportunities. Front. Cell. Infect. Microbiol..

[23] Kyathanahalli C., Snedden M., Hirsch E. (2023). Is human labor at term an inflammatory condition?. Biol. Reprod..

[24] Yellon S.M. (2020). Immunobiology of Cervix Ripening. Front. Immunol..

[25] Norwitz E.R., Greenberg J.A. (2009). Antibiotics in pregnancy: Are they safe?. Rev. Obstet. Gynecol..

[26] Jianu C., Rusu L.-C., Muntean I., Cocan I., Lukinich-Gruia A.T., Goleț I., Horhat D., Mioc M., Mioc A., Șoica C. (2022). In Vitro and In Silico Evaluation of the Antimicrobial and Antioxidant Potential of Thymus pulegioides Essential Oil. Antioxidants.

[27] Nadgir C.A., Biswas D.A. (2023). Antibiotic Resistance and Its Impact on Disease Management. Cureus.

[28] Moleriu L., Jianu C., Bujanca G., Doros G., Misca C., Ilie O.C., Moleriu R.D., Ilie A.C. (2017). Essential Oil of Hypericum perforatum. The chemical composition and antimicrobial activity. Rev. Chim..

[29] Cantarutti A., Rea F., Franchi M., Beccalli B., Locatelli A., Corrao G. (2021). Use of Antibiotic Treatment in Pregnancy and the Risk of Several Neonatal Outcomes: A Population-Based Study. Int. J. Environ. Res. Public Health.

[30] Mancuso G., Midiri A., Gerace E., Biondo C. (2021). Bacterial Antibiotic Resistance: The Most Critical Pathogens. Pathogens.

[31] Martinez de Tejada B. (2014). Antibiotic use and misuse during pregnancy and delivery: Benefits and risks. Int. J. Environ. Res. Public Health.

[32] Kakolwa M.A., Woodd S.L., Aiken A.M., Manzi F., Gon G., Graham W.J., Kabanywanyi A.M. (2021). Overuse of antibiotics in maternity and neonatal wards, a descriptive report from public hospitals in Dar es Salaam, Tanzania. Antimicrob. Resist. Infect. Control.

[33] Noda-Nicolau N.M., Tantengco O.A.G., Polettini J., Silva M.C., Bento G.F.C., Cursino G.C., Marconi C., Lamont R.F., Taylor B.D., Silva M.G. (2022). Genital Mycoplasmas and Biomarkers of Inflammation and Their Association with Spontaneous Preterm Birth and Preterm Prelabor Rupture of Membranes: A Systematic Review and Meta-Analysis. Front. Microbiol..

[34] Nguyen Q.H.V., Le H.N., Ton Nu V.A., Nguyen N.D., Le M.T. (2021). Lower genital tract infections in preterm premature rupture of membranes and preterm labor: A case-control study from Vietnam. J. Infect. Dev. Ctries.

[35] Tedesco R.P., Galvão R.B., Guida J.P., Passini-Júnior R., Lajos G.J., Nomura M.L., Rehder P.M., Dias T.Z., Souza R.T., Cecatti J.G. (2020). The role of maternal infection in preterm birth: Evidence from the Brazilian Multicentre Study on Preterm Birth (EMIP). Clinics.

[36] Daskalakis G., Psarris A., Koutras A., Fasoulakis Z., Prokopakis I., Varthaliti A., Karasmani C., Ntounis T., Domali E., Theodora M. (2023). Maternal Infection and Preterm Birth: From Molecular Basis to Clinical Implications. Children.

[37] Dorado Criado M., Fabra Garrido C., Merino San Martín E., González Arboleya C., Gómez-Arroyo B., González-Donapetry P., Baquero-Artigao F., de la Calle M., Quiles-Melero I., Calvo C. (2021). Is an Antenatal Screening for *Chlamydia trachomatis* Necessary in the Current Society?. Pediatr. Infect. Dis. J..

[38] Chelkeba L., Fanta K., Mulugeta T., Melaku T. (2022). Bacterial profile and antimicrobial resistance patterns of common bacteria among pregnant women with bacteriuria in Ethiopia: A systematic review and meta-analysis. Arch. Gynecol. Obstet..

[39] Nguyen M.H., Fornes R., Kamau N., Danielsson H., Callens S., Fransson E., Engstrand L., Bruyndonckx R., Brusselaers N. (2022). Antibiotic use during pregnancy and the risk of preterm birth: A population-based Swedish cohort study. J. Antimicrob. Chemother..

[40] Samarra A., Esteban-Torres M., Cabrera-Rubio R., Bernabeu M., Arboleya S., Gueimonde M., Collado M.C. (2023). Maternal-infant antibiotic resistance genes transference: What do we know?. Gut. Microbes..

[41] Tong H., Heuer A., Walker N. (2023). The impact of antibiotic treatment for syphilis, chlamydia, and gonorrhoea during pregnancy on birth outcomes: A systematic review and meta-analysis. J. Glob. Health.

[42] Mitchell C.M., Srinivasan S., Ma N., Reed S.D., Wu M.C., Hoffman N.G., Valint D.J., Proll S., Fiedler T.L., Agnew K.J. (2021). Bacterial Communities Associated with Abnormal Nugent Score in Postmenopausal Versus Premenopausal Women. J. Infect. Dis..

[43] Ligozzi M., Bernini C., Bonora M.G., De Fatima M., Zuliani J., Fontana R. (2002). Evaluation of the VITEK 2 system for identification and antimicrobial susceptibility testing of medically relevant gram-positive cocci. J. Clin. Microbiol..

[44] (2016). Performance Standards for Antimicrobial Susceptibility. Twenty-Second Informational Supplement.

[45] Walani S.R. (2020). Global burden of preterm birth. Int. J. Gynaecol. Obstet..

